# Male prisoners’ experiences of taking part in research about suicide and violence: a mixed methods study

**DOI:** 10.1186/s40900-021-00303-z

**Published:** 2021-09-14

**Authors:** Laura Hemming, Daniel Pratt, Gillian Haddock, Peer Bhatti, Jennifer Shaw

**Affiliations:** 1 Division of Psychology and Mental Health, School of Health Science, University of Manchester, Manchester, UK; 2 Manchester Academic Health Sciences Centre, Manchester, UK; 3 Greater Manchester Mental Health NHS Foundation Trust, Manchester, UK

**Keywords:** Suicide, Violence, Participation, Patient public involvement

## Abstract

**Background:**

There is an apparent reluctance to engage ‘vulnerable’ participants in conversation about sensitive topics such as suicide and violence and this can often lead to a paucity of research in these areas. This study aimed to explore the experiences of male prisoners taking part in quantitative and qualitative research on suicide and violence.

**Methods:**

Participants at four male prisons completed a visual analogue scale of mood before and after data collection for both a cross-sectional study and also a qualitative interview. Participants were also asked to give three words to describe their experience of participation. A paired samples T-test was conducted to explore the difference in pre- and post-mood ratings, and content analysis was conducted to explore the positive and negative comments on participants’ experiences.

**Results:**

Overall, participants’ mood significantly improved after participating in a cross-sectional study about suicide and violence (from 4.8 out of 10 to 5.3, *p* = 0.016), and there was no significant change in mood following participation in a related qualitative study (5.1 to 5.0, *p* = 0.793). Participants primarily described their experiences as positive, stating that the process had been satisfying, calming, interesting, enlightening and beneficial. A smaller number of participants described their experiences as stressful, challenging, saddening, uncomfortable and bizarre.

**Conclusions:**

This study has found that researching sensitive topics such as suicide and violence with male prisoners did not have a negative impact on mood, rather that participants largely enjoyed the experience. These findings dispel the myth that research about sensitive topics with prisoners is too risky and could inform how future researchers assess levels of risk to participants.

**Supplementary Information:**

The online version contains supplementary material available at 10.1186/s40900-021-00303-z.

## Plain English summary

Some topics of research are considered ‘sensitive’ or ‘risky’. This includes topics such as suicide and violence. Some groups of people are also considered ‘sensitive’ or ‘risky’. This includes prisoners. Because of this, there is little research done with prisoners on topics such as suicide and violence. This study aimed to explore the experiences of prisoners who took part in a study about suicide and violence. Eighty male prisoners took part in either a questionnaire study or an interview study (or both). At the beginning of the session people were asked to rate their mood from 0 to 10. Zero was the worst possible imaginable mood and 10 was the best possible imaginable mood. People were asked to do this again at the end of the session. People were also asked to give three words to describe their experience of the session. The people who took part in the questionnaire study had a significant improvement in mood after the session. The people who took part in the interview study had no difference in mood after the session. Most people gave positive words to describe the session. People said the session was satisfying, calming, interesting, enlightening and beneficial. A smaller number of people gave negative words. They described the session as stressful, challenging, saddening, uncomfortable and bizarre. This study therefore contrasts the idea that prisoners will become upset if they take part in research about suicide and violence. Therefore, we should continue to research this area.

**Supplementary Information:**

The online version contains supplementary material available at 10.1186/s40900-021-00303-z.

## Introduction

The declaration of Helsinki is one of the most important documents in research ethics [1]. The declaration has evolved over time from a simple statement of ethical code to a more prescriptive document [2]. In its most recent iteration, the World Medical Association notes the importance of the risk of harm that can arise from research participation, and states that *“medical research involving human subjects may only be conducted if the importance of the objective outweighs the risks and burdens to the research subjects”* [3]. Further, the declaration states that some groups and individuals are *“particularly vulnerable and may have an increased likelihood of being wronged or of incurring additional harm”*, and such groups should receive *“specifically considered protection”* [3].

Some researchers have come to consider specific topics of research and / or specific groups of participants as particularly open to the risk of harm from research participation. For instance, some have questioned the studying of topics such as suicide due to the psychological harms that may be associated with this. Indeed, researchers have reported concerns prior to embarking upon suicide research about the distress that may be caused to participants [4] with one survey finding that 65% of ethics committee members expressed the view that suicidal feelings or behaviours may be increased by research participation [5].

Despite this, there is now a body of evidence to suggest that, not only does taking part in suicide research not cause harm, it may also have a beneficial effect for participants. For example, a recent meta-analysis found that exposure to suicide-related content did not result in significant changes in levels of distress from pre- to post-assessment, nor did it lead to higher levels of immediate or delayed distress in comparison to those not exposed to content on suicide [6]. Furthermore, the same review found that there were small significant *reductions* in levels of suicide ideation from pre-to post-assessment and participants exposed to suicide-related content were significantly *less* likely to report a suicide attempt than individuals who were not exposed to such content [6]. Furthermore, qualitative studies have found that participants of suicide-related studies reported participation being therapeutic and cathartic and were grateful for an opportunity to reflect on past experiences and increase understanding [7]. Participants also reported that a key motivation for taking part was the notion that this could benefit others [7].

A particular group of participants that may be considered as ‘vulnerable’ are prisoners. Indeed, ethics review boards are known to label prisoners as “high risk” or “vulnerable subjects”, which can make access for researchers difficult and may hinder scientific inquiry [8]. Prisoners may be considered vulnerable due to the higher prevalence of physical and mental health problems than that of the general population [9–11], impacts of the prison environment such as social exclusion [10] and higher morbidity rates [12]. Others have raised concerns that prisoners may be more vulnerable to coercion or may lack the ability to give informed consent to participate in research [13]. Such concerns have led to a paucity of research being conducted with prisoners, due to fears about the vulnerability of this group [8, 13, 14]. Despite this, there is a small amount of evidence to suggest that conducting research with prisoners does not cause undue harm, and may even benefit participants [15].

### The present study

The present study describes a research methods study nested within a larger study which has already been published elsewhere [16, 17]. The larger study comprises a qualitative interview study exploring male prisoners’ experiences of alexithymia and how these relate to suicide and violence [16] and a cross-sectional questionnaire-based study exploring the relationship between alexithymia, suicide, violence and dual harm in male prisoners [17].

Alexithymia can be defined as an inability to identify or describe feelings and is also related to an externally oriented thinking style [18]. Research has shown that such a phenomena is related to suicide and violence [19], though previous research has not explored this relationship in a male prisoner population. Suicide is defined in these larger studies according to the definition given by NICE: *“any act of self-poisoning or self-injury carried out by a person, irrespective of their motivation”* [20]. Violence is defined in these larger studies according to the definition given by NICE: *“Violence and aggression refer to a range of behaviours or actions that can result in harm, hurt or injury to another person, regardless of whether the violence or aggression is physically or verbally expressed, physical harm is sustained or the intention is clear.”* [21]. Dual harm is defined in these larger studies as *“persons displaying both harm to self and harm to others”* [22].

The present study aimed to investigate male prisoners’ experiences of participating in both a qualitative enquiry and also a cross-sectional, questionnaire-based study that explored sensitive topics such as suicide, self-harm and violence.

## Methods

### Study design

The present study describes a research methods study nested within a larger study which has already been published elsewhere [16, 17]. A qualitative structured interview was used in the present study. The larger study from which this sub-study derives used mixed methods including semi-structured qualitative interviews and structured questionnaires.

### Participant selection

Data for this study were taken from participants who had been selected to take part in either, or both, a qualitative study [16] or a cross-sectional study [17] exploring the relationship between alexithymia and suicide, violence and dual harm. Data for the current study were collected during the data collection sessions for each of these other studies [16, 17]. Participants were identified as eligible to participate in the cross-sectional study if they had been identified either by a member of prison staff or via self-report as having recently engaged in an actual or expressed suicide attempt or act of violence, or were deemed at risk of engaging due to ideation. A subset of participants from this study, identified as having high levels of alexithymia according to a score of 52 or above on the Toronto Alexithymia Scale (TAS-20) [23], were invited to participate in a qualitative study. Participants were approached by a researcher and provided an information sheet and were given at least 24 h to consider their participation in these studies.

### Setting

Data were collected from three host prison sites in the North West of England. These included a category ‘A’ prison (maximum security), a category B prison (establishments for those who do not require maximum security but for whom escape must be made difficult) and a category C prison (for prisoners who cannot be housed in open conditions but who are unlikely to try to escape). Data collection occurred in a private room within each of the prisons, with only the researcher and participant present.

### Patient and public involvement

A patient and public involvement group was recruited to assist with all studies, in order to ensure data collection, analysis and dissemination included the views of individuals with lived experience of incarceration. The group inputted to the design, the data collection tools (including quantitative questionnaires and qualitative topic guides) and made important changes to the interview process which highlighted the need to recognise participant concerns and distress. A full outline of the extent of patient and public involvement is outlined elsewhere [24] and is also detailed in the GRIPP2 Short Form Checklist (Additional file 3). Specific to the current study, an individual with lived experience of incarceration was consulted on the analysis and paper writing stages.

### Data collection

For detailed descriptions of the full data collection procedures for all studies, please see previous papers [16, 17]. For the current study, participants taking part in both the qualitative and the cross-sectional studies were asked to complete a visual analogue scale (VAS) [25] (ranging from 0 ‘worst imaginable mood’ to 10 ‘best imaginable mood’) before and after each data collection session (Additional file 1: Appendix A). A visual analogue scale was chosen due to it providing a well-validated and established tool, its ease in administration and acceptance by respondents [26]. Moreover, using a visual analogue scale for mood has been found to have test-restest reliability, as well as concurrent validity with respect to depression [27]. At the end of each data collection session, participants were asked: *“If you could choose any three words to describe our session today, what would they be?”*. Participants were encouraged to provide their own words, but for those who struggled or showed a reluctance to do so, a list of possible words (Additional file 2: Appendix B) was given for participants to choose from, informed by previous research on the experiences of participating in suicide research as well as discussions with a patient and public involvement group. The researcher then asked the participant to provide further context and reasoning for each word chosen, and extensive notes were made based on participant’s responses. All interviews were conducted by the lead author (LH) who is a female PhD student in her late twenties, with a background in clinical psychology.

### Ethical and safety considerations

Given the sensitive nature of this study, important considerations were taken to ensure the safety both of participants and the researcher. Where participants' visual analogue mood scores decreased from pre- to post-data collection, the interviewer asked follow-up questions to the participant to ascertain the level of distress or risk of harm to self and/or others. Where this was considered to be high, a distress protocol was followed whereby any information necessary was passed onto relevant prison staff and recorded in relevant logbooks.

LH had received all necessary security clearances, including a DBS certificate, to enable her to travel freely around the prison. LH completed an induction at each of the host prisons which informed her of how to remain safe whilst in prison, and included knowledge of how to raise an alarm if necessary.

### Data analysis

#### Statistical analysis

All statistical analyses were conducted using IBM SPSS version 25 for Windows. Responses from participants in the qualitative study and cross-sectional study were separated in order to avoid violating the assumption of independence of observations. The pre- and post-VAS data were checked for normality, via visual inspection of histograms. Paired samples T-tests were conducted to ascertain whether there was a significant difference in mood scores before and after data collection sessions.

#### Content analysis

First, a list of words used to describe data collection sessions was compiled across both the qualitative and cross-sectional studies, with frequencies of each word recorded. Word clouds were created to illustrate visually the frequency of words given, using a free online word cloud generator (https://www.wordclouds.co.uk/). Following this, the words were sorted into ‘positive’, ‘neutral’ and ‘negative’ words by LH and PB. Words were categorised according to the word itself, as well as the accompanying free text which helped to give context to whether the word was used in a positive, neutral or negative manner. Only positive and negative words are reported upon in this paper. Within this framework, similar words were then grouped together to form themes, which were reviewed and revised following discussion between authors, including an individual with lived experience of incarceration. Where notes had been made about the context of each word, these were analysed using thematic analysis [28].

## Results

### Changes in mood

A total of 79 (out of 80) participants provided pre- and post-VAS scores when completing the cross-sectional data collection. The participants were male, predominantly White British (87%) and had a mean age of 34.7 years (SD = 9.02). The pre- and post-VAS data for cross-sectional participants were considered to be normally distributed. The mean score for mood at the beginning of cross-sectional data collection sessions was 4.8 (SD = 2.2) and the mean score for mood at the end of the session was 5.3 (SD = 2.0). A paired samples T-test revealed this to be a statistically significant improvement in mood (*t* = − 2.47, *p* = 0.016 (two-tailed)).

A total of fourteen (out of fifteen) participants provided responses about their experiences of participating in the qualitative study. The participants were male, predominantly White British (86%) and had a mean age of 34.4 years (SD = 8.99). The assumption of normal distribution was considered to be met. There was no significant difference in pre-VAS scores (5.1, SD = 2.1) and post-VAS scores (5.0, SD = 2.5) for the qualitative study (*t* = 0.27, *p* = 0.793 (two-tailed)).

### Thematic and content analysis

**Fig. 1:**
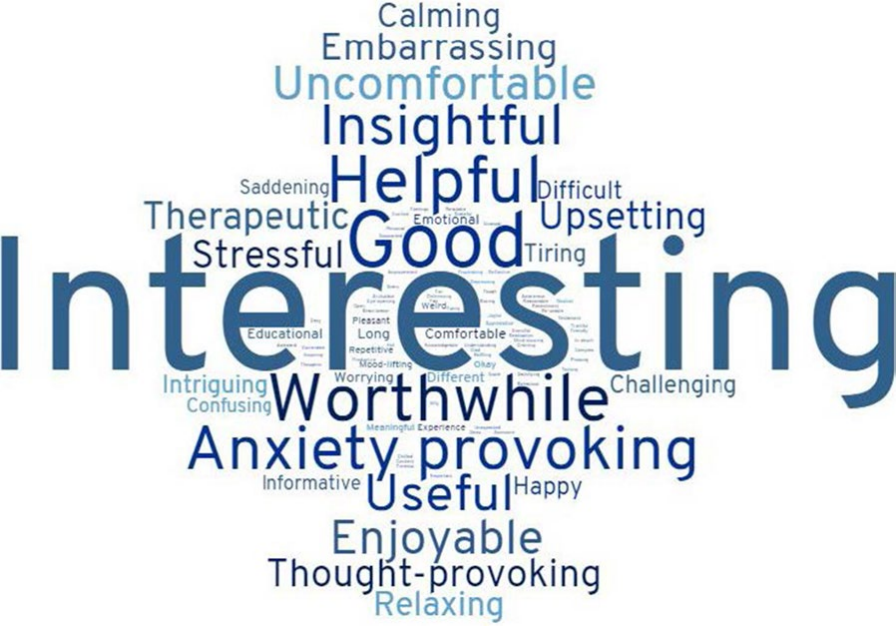
Word cloud of all words given

A total of fourteen participants provided feedback about their experience of participating in the qualitative study (fifteen participants in total) and 77 participants provided responses about their experiences of participating in the cross-sectional study (80 participants in total). Across all 91 participants, a total of 97 words were given, and these words were given a total of 323 times. A total of sixteen words (16%) were from the list of suggested words and these words were mentioned a total of 167 (52%) times. Figure 1 shows all words chosen by participants, with larger words representing those chosen more frequently. The words most frequently given were interesting (N = 39), good (N = 16), worthwhile (N = 14) and helpful (N = 14).

#### Positive words

**Fig. 2:**
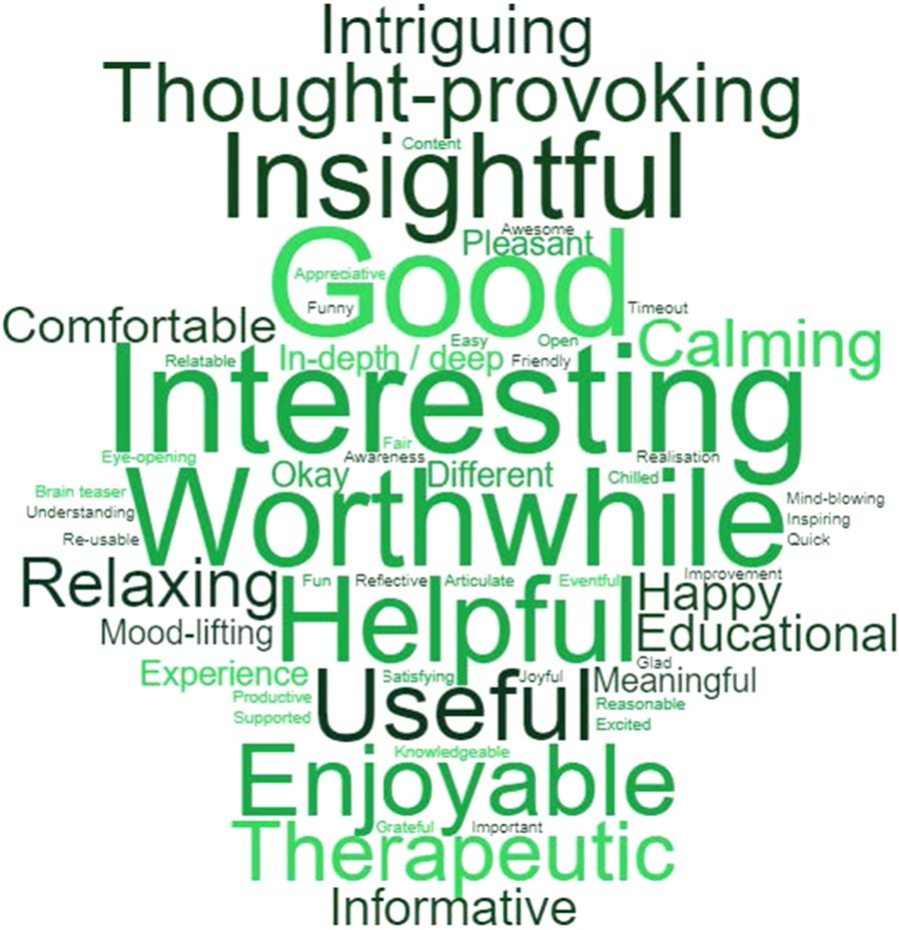
Word cloud of positive words given

Of the 97 words provided, a total of 58 (60%) words were coded as positive, and these were mentioned a total of 216 times (67%). A total of 43 participants used only positive words to describe the session, and a further 30 participants used both positive and negative words to describe the session. The most frequently used positive words were interesting (N = 39), good (N = 16), worthwhile and helpful (N = 14). Figure 2 shows all positive words chosen by participants, with larger words representing those chosen more frequently. The 58 positive words were grouped into seven themes; satisfying, calming, interesting, enlightening, beneficial, a new experience and positive words to describe the researcher and research process. Each of these themes will be expanded upon in the sections below.

##### Satisfying

Participants used a range of words to describe how the data collection sessions had been satisfying including good (N = 16), enjoyable (N = 10), happy (N = 5), pleasant, mood-lifting, okay (N = 2), awesome, fun, funny, content, joyful and satisfying (N = 1). Primarily participants described the session as ‘good’ due to the opportunity allowing them to discuss feelings, get things off their chest and have somebody listen to them.

Someone listening. It helps. Normally it falls on deaf ears. (W45)

Good. To. Talk. It’s nice to say how it is to somebody like you. (W46)

People also described how they had enjoyed the session, had had fun, and found some of it funny even. This led to participants feeling content and happy and participants described their mood improving after completing the session.

Some of the questions were fun and we had a laugh. (W48)

I’d do it again. I feel better now that I’ve done it. (W60)

##### Calming

As well as describing the sessions as satisfying, participants described the sessions as relaxing, calming (N = 7) and chilled (N = 1). Again, this was associated with an improvement in mood following data collection.

I felt relaxed for the whole thing even though some of the questions were a bit touchy, I tried to answer all of them honestly. (W61)

##### Interesting

Participants described the sessions as interesting (N = 39), intriguing (N = 5), exciting, relatable and even mind-blowing (N = 1). Participants tended to be interested in the types of questions being asked of them, interested to find out more about themselves through the process of data collection and also interested by how relatable the questions felt to them.

The questions related to me. I’m interested in how you’ve come about those questions. I felt it was directed to me. (W28)

##### Enlightening

Participants spoke about data collection sessions being enlightening and used a range of words to describe this; insightful (N = 12), thought-provoking (N = 8), informative (N = 4), educational (N = 3), productive, articulate, reflective, awareness, improvement, knowledge, realisation, brain teaser and eye-opening (N = 1). Primarily, people spoke about the data collection session inviting them to think about things they hadn’t previously considered or reported that they saw things in a different light to what they had previously.

Making me think about what sort of person I am and what I need to be when I go through the gates. (W53)

Learning something about myself and understanding my own thoughts and feelings. (W41)

Emotional improvement – I don’t tend to understand emotions, but now I understand them a little bit more. (W55)

##### Beneficial

Participants described the sessions as being beneficial both to themselves and to others in a range of ways. Participants used the following words to describe these benefits: helpful, worthwhile (N = 14), useful (N = 11), therapeutic (N = 8), comfortable (N = 3), meaningful (N = 2), important, inspiring, appreciative, glad, grateful, re-usable and supported (N = 1). People spoke about the benefits of participating for themselves being mainly that the session had been cathartic or therapeutic for them, that they had gained knowledge from taking part or that their mood had improved as a result of taking part.

It makes me think more. Although I was told there will be no direct benefit for taking part, I feel that I am getting something out of it just by doing it. (M08)

In addition, participants stressed that they were glad to have taken part due to it being helpful to others, including the researcher and also others in the future who may find themselves in a similar situation to the participant.

I feel it’s helping someone else down the line. (W15)

Because it will help the researcher to obtain PhD and help others in the future who are feeling suicidal. (M04)

##### A new experience

Participants spoke about taking part in research as being a new experience which was viewed as a welcome break from the monotony of prison life. Participants used words to describe this such as different (N = 3), experience (N = 2), timeout and eventful (N = 1).

Not something I do every day, or every week, or every month. It was a good difference because it got me out of my cell. (W30)

##### Positive words to describe the researcher and research process

Participants described the researcher as friendly (N = 1), open (N = 1) and understanding (N = 1). Participants viewed these traits positively.

Researcher was patient and smiley. (W35)

Participants described the research process as in-depth/deep (N = 2), easy, fair, quick and reasonable (N = 1). Again, these were all viewed favourably by participants:

I enjoyed going as in-depth with questions. It’s different to anything I’ve done before. (W40)

You get all the options to the answers and the questions seem reasonable. (W36)

##### Negative words

**Fig. 3:**
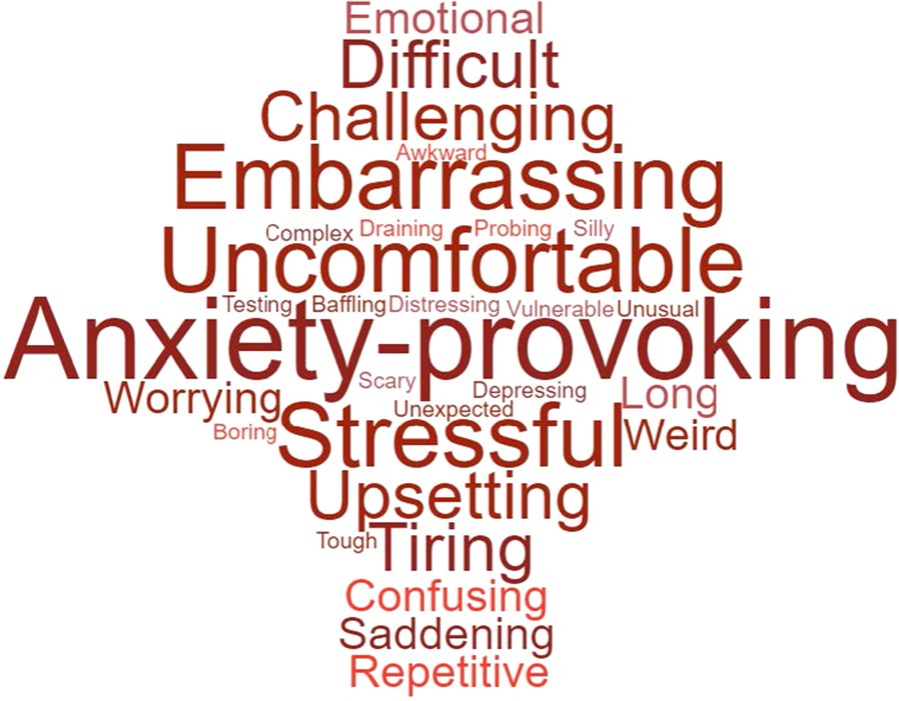
Word cloud of negative words given

Of the 97 words given in total, participants used a total of 30 (31%) negative words to describe the sessions with a total frequency of 98 (30%). Ten participants gave negative words only to describe the sessions, and a further 30 participants gave a combination of positive and negative words to describe the sessions. The most frequently used negative words were anxiety-provoking (N = 13), uncomfortable (N = 10), stressful, embarrassing, and upsetting (N = 8). Figure 3 shows all negative words chosen by participants, with bigger words representing words chosen more frequently. The 30 negative words given were grouped into seven themes: stressful, challenging, saddening, uncomfortable, dull, bizarre and a complicated process. Each of these themes will be expanded upon in the sections below.

##### Stressful

Some participants described data collection sessions as anxiety-provoking (N = 13), stressful (N = 8), worrying (N = 3), scary and distressing (N = 1). People spoke about being worried due to not knowing what to expect and finding answering the questions anxiety-provoking. It is worth noting that several participants identified this as due to having existing anxiety problems, and others mentioned that the anxiety dissipated as the session went on.

Sometimes answering the questions truthfully was anxiety provoking. (W11)

Once the questions started, I wasn’t anxious. But at the start some questions made me feel uncomfortable. (W25)

At times distressing due to some of the more intense questions, e.g. about suicide. (W08)

##### Challenging

Participants spoke about finding participating emotionally challenging using words such as difficult, challenging (N = 5), testing, tough and probing (N = 1) to describe the process. This was typically related to questions reminding participants of past difficult times, and it being difficult for participants to re-experience this.

I had to think about things, but I enjoyed the challenge. (G01)

As a result of this emotional demand, some participants described the session as tiring (N = 5) and draining (N = 1).

##### Saddening

In contrast to participants describing the session as satisfying (3.2.1.1), some participants spoke about sessions being upsetting (N = 8), saddening (N = 4), emotional (N = 3) and depressing (N = 1). People particularly felt that questions about suicide and self-harm could be upsetting.

It’s made me feel a bit sad. (W39)

##### Uncomfortable

Despite participants reporting feeling comfortable with the sessions, some reported feeling uncomfortable (N = 10), embarrassed (N = 8), awkward and vulnerable (N = 1). Participants largely cited reasons for this discomfort as being due to the personal nature of the questions, and also cited specific reasons such as being embarrassed by a lack of literacy and being asked about sexual appetite and their offence.

Getting started was quite uncomfortable because they’re quite personal questions. Once we got started I felt comfortable. (W60)

##### Dull

Some participants described the session as long (N = 3), repetitive (N = 2) and boring (N = 1). Particularly in relation to the cross-sectional study, participants felt that they were often asked the same questions in different ways, and this led to them becoming disinterested in the process.

Lost interest because it took too long. (M09)

##### Bizarre

A very small number of participants described sessions as weird (N = 2), silly, unexpected and unusual (N = 1). This was mainly in relation to participants feeling ‘weird’ about opening up to a relative stranger, and also was about unknown aspects of the assessment.

You’re new to me, so it’s hard to try and answer the questions you’ve asked me. (W56)

Not knowing what was going to be asked. (W19)

##### Complicated process

In direct contrast to those participants who described the session as easy and fair, others described it as confusing (N = 4), complex and baffling (N = 1). This was typically in relation to being asked questions they hadn’t previously considered, and due to the diversity and breadth of questions asked.

A lot of questions and lots of different answers. It’s not straightforward, but it’s manageable. (W59)

## Discussion

This study has found that overall, participating in a research study about sensitive topics including suicide and violence had positive benefits. Although there were some negative impacts, these were less frequent than positive, and overall, mood improved following participation. Whilst there was no significant change in mood following participation in a qualitative study, it is important to note that the T-test included only fourteen participants, so it is likely that there was not enough power to detect a significant change in mood. The findings of this study echo previous findings of a meta-analysis which found that participating in suicide research did not lead to a statistically significant increase in distress [6]. Pertinent to this sample, previous suicide research with male prisoners [15] found a significant increase in mood scores using a VAS from 4.0 pre-session to 5.0 post-session which is in line with the findings reported in this study. This study therefore contributes to a body of literature which suggests that researching sensitive topics such as suicide amongst male prisoners may be beneficial to the mood of participants.

The words chosen to describe experiences of participation were predominantly positive with participants describing their participation as satisfying, calming, interesting, enlightening and beneficial. Participants also described the research process and the researcher positively and acknowledged that participating had given them an opportunity to experience something new. Previous studies have found that ‘vulnerable’ participants have described participating in the research process as beneficial both to themselves and to others [7, 15, 29, 30]. Others have noted the educational aspects of participating in research about sensitive topics, which has led to participants feeling enlightened [7, 29–32]. Some have also reflected the findings in this study that participants found the research process enjoyable, fun and good [7, 29, 30]. Furthermore, previous studies have reported participation had a calming, therapeutic effect on participants which is in line with the findings of the current study [15, 29]. Pertinent to this sample, one study [15] previously found that a key positive aspect of research participation for prisoners was the opportunity for time out of their cell, which is again reflected in the current findings.

Despite the overwhelmingly positive experiences of participation, it is important to acknowledge that some participants described their experiences negatively. Namely, participants found the experience to be stressful, challenging, saddening, uncomfortable, bizarre and complicated. It is important to note, however, that participants who gave negative words did so most frequently in combination with positive words, indicating that very few participants found the process to be entirely negative. In addition to this, many of the responses accompanying the negative words given outlined that although there may have been negative aspects of participation, these were deemed to be acceptable by participants. Previous studies have echoed findings that participants may experience initial apprehension or nerves prior to participation [7, 29, 30, 32]. Moreover, it has commonly been reported that discussing suicide in the research context can be experienced as distressing by participants, but that this distress is both transient and manageable [7, 29–31]. Pertinent to this sample, one previous study [15] also found that male prisoners described the research questions as silly, confusing and probing which is directly in line with the findings reported here.

### Lived experience reflections

As aforementioned, an individual with lived experience of incarceration was invited to contribute to this paper at a number of different stages. In this section, PB outlines his reflections on this experience, providing an overview of how the findings of this study resonate with his own lived experience of incarceration.

My comments are based on my lived experience as an ex-prisoner. The findings reflect my experiences of prison life and of others I came into contact with. I can relate to the comments made, for example that being involved in the study breaks up the monotony of prison life. But more than that, it offers an opportunity to engage with something perhaps new to the participant such as the field of research and education. It may offer an introduction to a topic they may never have considered or knew existed, but they may notice a connection to how they view the world and themselves (e.g. with alexithymia in this case).

The findings appeared to be that participants felt valued and felt that their participation gave them back some self-worth which may have been eroded. Prison can sometimes feel like a zoo. The researcher can be seen as the one doing a field study of animals (prisoners) that they may never have encountered face to face other than on TV, such as the more exotic animals (violent prisoners). This is therefore often outside of the researcher’s realm of experience and is like a field study. The zookeeper (prison staff) may tell the researcher to watch out, be careful, they’re dangerous animals, don’t turn your back for a second and pay attention to the safety briefing. However, participants in this study described feeling like they were being asked for their help, and not being studied like an ‘exhibit’. They may have felt useful being able to contribute to research which may help others in the future. People like to feel needed, and these participants might have been instrumental in bringing about positive change. Comments about wanting to help the researcher with their PhD suggested that they felt needed by someone else rather than them having to seek someone else's approval or needing something themselves and having to submit to authority.

The language used by participants felt participatory and inclusive. Participants acknowledged that it had been outlined to them that there were no direct benefits to taking part, but still they felt they had benefited from the fact they had been asked to take part. They had been consulted. This outlines to me that the participants may have been used to having things done *to* them as opposed to *with* them and this felt less of a disempowering experience.

Participants used very positive language throughout about their experiences, but this could be due to the artificial environment of being contained and them feeling obligated, or even conditioned, to give positive favourable responses, and not to rock the boat. They may have been conscious of appearing to be cooperating to look good on their record of being a good participant, and to avoid disappointing the researcher. Alternatively, they may have felt peer pressure or pressure from staff for example. They may have little contact with the outside world or no visitors and having interaction with someone may have therefore increased feelings of well-being.

The results showed that taking part did not lead to a decrease in mood, although this finding was not statistically significant for those who participated in the qualitative study. I think the qualitative interviews were more challenging as these can potentially lead to participants feeling they have opened up a can of worms by discussing sensitive topics such as violence/suicide. This may lead to participants reflecting upon the pertinence of the questions asked and puzzling over themes that appear outside of their realms of experience. Hence why they commented they found aspects 'bizarre'.

There can be triggering words, phrases and scenarios which can be more hard hitting than when rating mood using scales. Also, any harms associated with participating may not be obvious to the researcher, or the participant, until later. For instance, participation in the study may not be instantly recognisable as a trigger for something and could lead to a decline in mood that the participant is not aware of.

I think the results demonstrate the need for an advisory group to be involved in planning of any future research from the outset to mitigate these factors. However, it’s important to be mindful of the training issues such as issues around prior knowledge, education, power imbalances around research/ academia and issues of class, race, poverty and disadvantage amongst other factors. Also access needs such as documents in large print and a glossary of terms used which may be unfamiliar to participants, should be considered when working with advisory group members.

### Strengths and limitations

This study makes a unique contribution to the literature in its exploration of male prisoners’ experiences of taking part in research on sensitive topics such as suicide and violence. The study has benefitted from exploring in some detail individuals’ experiences whilst adopting a relatively brief methodology, which avoided placing undue burden on participants or engaging in an arduous process. The study also benefits from its inclusion of an individual with lived experience of incarceration, which helped to contextualise the words given by participants and to provide a different perspective on the findings. A final strength of this study lays in its diversity of host prisons, spanning several different categories and functions, meaning that these prisons therefore represent prisons typical of the UK.

Despite this, the study is not without limitations. The comments given by participants were verbal, and notes were written by the researcher. This may have led to inaccuracies for example in comparison to having recorded conversations and transcribing them verbatim; interpretation or meaning may have been altered between the participants’ comments and the researcher’s recording of these. Moreover, this brief methodology inhibits the opportunity to explore in-depth individual’s experiences of participating in sensitive research, since data is limited to short excerpts only. Related to this, this sample represents a particularly challenging sample to conduct this nature of research with; indeed, several participants had high scores on a measure of alexithymia indicating difficulties with describing feelings. Thus, whilst it may have been necessary to provide a predetermined list of words to choose from, this somewhat inhibits the ability for participants to describe their feelings, and may have introduced bias. It is also possible that participants may have been reluctant to give honest opinions about the research experience, given that the same researcher completed the data collection with them and then asked about their experiences of this. Despite concerted efforts to recruit a diverse range of participants, this sample was predominantly White British, and it is important to note that other populations may respond differently to these sensitive topics for instance females, LGBTQIA+ and BIPOC. Finally, the data were collected immediately after the data collection session ceased; it is therefore not possible to draw conclusions about the long-term consequences of participation or whether the positive and negative impacts described here were lasting.

### Future research and implications

Future research should aim to explore in greater detail male prisoners’ experiences of participating in sensitive research. Specifically, future research should utilise more open qualitative methodologies (such as qualitative interviews using semi-structured topic guides), and responses should be audio recorded and transcribed verbatim. Future researchers should also consider appointing a third party, possibly a researcher with lived experience, to independently conduct the research to minimise the potential impact of social desirability. Future researchers should also consider exploring similar topics in a more diverse range of prison populations, for instance youth offenders, female prisoners and prisoners from BIPOC communities.

The findings of this study also have important implications for those researching sensitive topics with ‘vulnerable’ populations. This study has shown that conducting research with male prisoners on sensitive topics did not impact negatively on mood, and in fact improved participants’ moods, on average. Future researchers and ethics review boards should therefore consider this evidence when reviewing research topics and methodologies and use the evidence to make a balanced assessment about the likelihood of participants experiencing harm as a result of participating in such studies.

Others have noted the difficulties that researching in prisons can bring [33–35], and this, combined with a perception of prisoners as a ‘vulnerable’ population, has led to a paucity of research with this population [8, 13, 14]. However, this study has found that not only does research with such a population contribute to both clinical and scientific improvements, but it may also provide benefits to the participants themselves. Specifically, participants reported feeling educated or enlightened as a result of taking part, and also found that their mood improved as a result of participation. Researchers should therefore become more confident when conducting research with this population who are often considered ‘vulnerable’.

Lastly, the results of this study can also be used to improve both recruitment rates to studies as well as to improve the experiences of participants who agree to take part. For instance, it may be appropriate to include material in participant information sheets which outlines some of the possible benefits of participating in research such as feeling calm and happy after participating, having an opportunity to experience something new (particularly relevant to prisoner populations) and the possible benefits both to the individual and to others. Moreover, the findings reveal some practical points which should be considered in the research process, for instance ensuring that researchers are open, friendly and understanding, and ensuring that research processes are fair, easy, quick and reasonable. Working with a patient and public involvement group may be of use with this latter point, to ensure that all questions being asked of participants are deemed appropriate and not too complicated. Furthermore, it would also seem appropriate to communicate to potential participants the possibility of negative consequences such as finding the experience stressful, challenging, saddening and uncomfortable. It would be worth explaining to participants that the majority of people find these effects to be transient and manageable [7] and do not outweigh the perceived benefits of taking part in sensitive research. Finally, there may be a need for researchers to consider the ordering of questions, such that those that inquire directly about experiences of suicide or suicidal thoughts are placed in the middle of assessments, to allow time to improve participants moods should they have lowered as a consequence of these questions.

## Conclusions

This study has found that male prisoners participating in a study about suicide and violence did not experience a significant negative impact on their mood. Moreover, participants generally described the experience of participating in a positive manner, with negative comments given mainly in tandem with positive comments. This study therefore suggests a need for researchers to reassess their views of prisoners as a ‘sensitive’ population, and also to reassess topics such as suicide and violence as ‘sensitive’ or ‘risky’. This is important, given the paucity of research in these populations on such topics.

## Supplementary Information

**Additional file 1.** Visual Analogue Scale of mood.

**Additional file 2.** List of suggested words.

**Additional file 3.** GRIPP2-SF.

## Data Availability

The dataset used during the current study are available from the corresponding author on reasonable request.
