# Supplementary material for: Male prisoners’ experiences of taking part in research about suicide and violence: a mixed methods study
Source: Res Involv Engagem. 2021 Sep 14;7:65. doi: 10.1186/s40900-021-00303-z (PMC8438986; doi:10.1186/s40900-021-00303-z)
Supplement: Supplementary file 1 — Additional file 1. Visual Analogue Scale of mood. [file 40900_2021_303_MOESM1_ESM.pdf]

MANCHESTER 1824

The University of Manchester

Visual analogue scale

Participant ID Number **Date:**

Instructions:

To help people say how happy or upset they feel, we have drawn a scale (rather like a thermometer) on which the best state you can imagine is marked 100 and the worst state you can imagine is marked 0.

We would like you to indicate on this scale how you feel at the moment. Please do this by drawing a line from the box below to whichever point on the scale indicates how good or bad your emotional state is now.

Your own emotional state at the moment

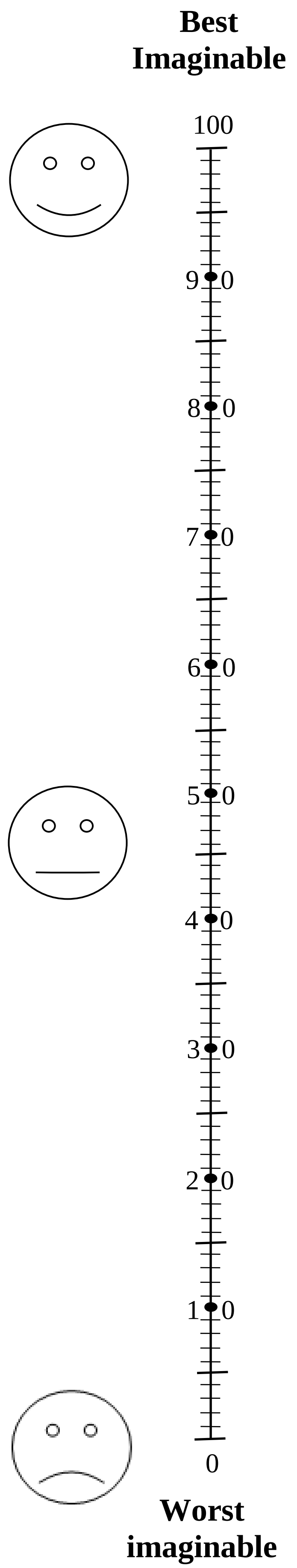

Version 1, 29.09.17
IRAS ID: 236345
