# Supplementary material for: Male prisoners’ experiences of taking part in research about suicide and violence: a mixed methods study
Source: Res Involv Engagem. 2021 Sep 14;7:65. doi: 10.1186/s40900-021-00303-z (PMC8438986; doi:10.1186/s40900-021-00303-z)
Supplement: Supplementary file 2 — Additional file 2. List of suggested words. [file 40900_2021_303_MOESM2_ESM.docx]

**End of Assessment Experiences of Participating in Suicide Related Research**

**Checklist of emotions attributed to research assessments**

*Participant ID: ___________ Date: ___/___/_____*

Circle all the adjectives below that are mentioned during the interview that relate to the experience of taking part in suicide research. Explore those not spontaneously covered by the participant.

- We’d like to know whether the experience of doing this assessment with us about suicidal thoughts and acts was……

| *The experience of talking to a researcher about suicidal thoughts and acts was….* | |
| --- | --- |
| Enjoyable | Interesting |
| Upsetting | Saddening |
| Stressful | Insightful |
| Tiring | Worrying |
| Uncomfortable | Therapeutic |
| Relaxing | Worthwhile |
| Calming | Embarrassing |
| Anxiety provoking | Useful |
| Any other: | |
