# Supplementary material for: Male prisoners’ experiences of taking part in research about suicide and violence: a mixed methods study
Source: Res Involv Engagem. 2021 Sep 14;7:65. doi: 10.1186/s40900-021-00303-z (PMC8438986; doi:10.1186/s40900-021-00303-z)
Supplement: Supplementary file 3 — Additional file 3. GRIPP2-SF. [file 40900_2021_303_MOESM3_ESM.docx]

# GRIPP2 short form

| Section and topic | Item | Reported on page no. |
| --- | --- | --- |
| 1: Aim | Report the aim of PPI in the study | 6 |
| 2: Methods | Provide a clear description of the methods used for PPI in the study | 6 |
| 3: Study results | Outcomes – report the results of PPI in the study, including both positive and negative outcomes | 17 |
| 4: Discussion and conclusions | Outcomes – comment on the extent to which PPI influenced the study overall. Describe positive and negative effects. | 19-21 |
| 5: Reflections / critical perspective | Comment critically on the study, reflecting on the things that went well and those that did not, so others can learn from this experience. | 19-21 |
